# Lymphocytic Infiltrate and p53 Protein Expression as Predictive Markers of Response and Outcome in Myelodysplastic Syndromes Treated with Azacitidine

**DOI:** 10.3390/jcm10214809

**Published:** 2021-10-20

**Authors:** Carlo Pescia, Francesca Boggio, Giorgio Alberto Croci, Ramona Cassin, Marco Barella, Loredana Pettine, Gianluigi Reda, Elena Sabattini, Carlo Finelli, Umberto Gianelli

**Affiliations:** 1 Division of Pathology, Fondazione IRCCS Ca’ Granda Ospedale Maggiore Policlinico, 20122 Milan, Italy; carlo.pescia@unimi.it (C.P.); francesca.boggio@policlinico.mi.it (F.B.); giorgio.croci@unimi.it (G.A.C.); marco.barella@unimi.it (M.B.); 2 Department of Pathophysiology and Transplantation, Università degli studi di Milano, 20122 Milan, Italy; 3 Division of Hematology, Fondazione IRCCS Ca’ Granda Ospedale Maggiore Policlinico, 20122 Milan, Italy; ramona.cassin@policlinico.mi.it (R.C.); loredana.pettine@policlinico.mi.it (L.P.); gianluigi.reda@policlinico.mi.it (G.R.); 4 Hematopathology Unit, IRCCS Azienda Ospedaliero-Universitaria di Bologna, 40138 Bologna, Italy; elena.sabattini@aosp.bo.it; 5 Division of Hematology “Seràgnoli”, IRCCS Azienda Ospedaliero-Universitaria di Bologna, 40138 Bologna, Italy; carlo.finelli@unibo.it

**Keywords:** myelodysplastic syndromes, azacitidine, bone marrow histology

## Abstract

High-risk Myelodysplastic syndromes (MDS) represent therapeutical challenges and are usually managed with hypomethylating agents such as azacitidine. Given the lack of data in the literature concerning azacitidine effects on bone marrow, we retrospectively analyzed 57 high-risk MDS cases in order to identify any changes induced by azacitidine therapy or relevant correlations between therapy response and pre- or post-treatment features. Azacitidine treatment had no significant impact on bone marrow cellularity or morphological dysplastic features. On the contrary, although not statistically significant, we observed a slight decrease in CD34+ and CD117+ blasts and p53+ precursors after treatment. Moreover, pre-treatment IPSS-R cytogenetic score (*p* = 0.004), lymphocytic infiltrate (*p* = 0.017) and p53+ elements (*p* = 0.001) correlated with AML progression; pre-treatment lymphocytic infiltrate was also linked to better response to therapy (*p* = 0.004), suggesting an anti-tumoral role of bone marrow microenvironment. Post-treatment blast count impacted negatively on overall survival (*p* = 0.035) and risk of leukemic progression (*p* = 0.04), while both post-treatment lymphocytic infiltrate and p53+ elements showed significant correlation with treatment response (*p* = 0.004 and *p* = 0.003 respectively). Higher post-treatment p53+ elements correlated also with risk of leukemic progression (*p* = 0.013). Our results suggest the possible role of lymphocytic infiltrate and p53+ elements as predictive markers in MDS treated with azacitidine, disclosing new chapters in the understanding of MDS evolution and treatment.

## 1. Introduction

Myelodysplastic syndromes (MDS) are a heterogeneous group of clonal myeloid neoplasms, characterized by peripheral cytopenia due to ineffective bone marrow hematopoiesis related to morphological dysplasia in one or more myeloid lineages. MDS frequently display recurrent genetic abnormalities and have a high risk of progression to acute myeloid leukemia (AML), which identifies them as pre-leukemic conditions [1,2,3]. MDS patients can be stratified using two different prognostic scoring systems, namely the International Prognostic Scoring System, the revised-International Prognostic Scoring System (IPPS-R) [4], and the WHO Classification-based Prognostic Scoring System (WPSS), which both predict survival and risk of AML evolution [5,6]. While low-risk MDS have a better prognosis and can be clinically managed with approaches varying from active surveillance to erythropoiesis-stimulating agents, current treatment of high-risk MDS, whose life expectancy is inferior to two years, is essentially based either on hypomethylating agents (decitabine, azacitidine) or intensive chemotherapy. Ideally, all high-risk MDS should be evaluated for allogenic stem-cell transplant, but in the majority of cases, patients’ age and performance status do not allow aggressive approaches [3]. Most high-risk cases are treated with hypomethylating agents, such as azacitidine, which guarantee hematological response in half of cases [7]. The rationale for treatment with this drug, which appears effective although not curative, is based on the observation that epigenetic modifications such as methylation and transcriptional regulation are frequent in MDS (and secondary AML) [8]. Features predicting a poor response of high-risk MDS to azacitidine are prior treatment with low-dose cytosine arabinoside, bone marrow blasts >15%, abnormal karyotype [9], and bone marrow fibrosis equivalent to or higher than MF-1 [10]. However, despite the wide usage of hypomethylating agents and especially of azacitidine in MDS, little is known about the morphological and immunophenotypical modifications induced by treatment on bone marrow cellularity, although some studies have focused on the effects of azacitidine on single cellular compartments, such as mesenchymal stem cells [11]. Silverman et al. [12] in 1993 observed a significant reduction in bone marrow erythroid progenitor cells assessed in vitro after azacitidine administration, with parallel amelioration of peripheral blood count and bone marrow blast count. A recent study [13] has focused on the impact of azacitidine on immunophenotypic features of bone marrow evaluated by means of flow cytometry, demonstrating that azacitidine improves flow cytometry values in about 40% of high-risk MDS patients, reducing the number of CD34+ blasts and restoring myeloid or monocytic pattern maturation in a smaller subset of cases. This immunophenotypic improvement proved to be linked to better clinical response. However, flow cytometry evaluation was not paired with bone marrow histological assessment. Hence, our main purpose was to evaluate bone marrow comprehensive features in patients with MDS before and after treatment with azacitidine, in order to assess the relevance of modifications induced by therapy. We also focused on the retrospective correlation between response to azacitidine and histological and immunophenotypic features of bone marrow biopsies, in order to evaluate putative predictive factors of response to hypomethylating agents.

## 2. Materials and Methods

### 2.1. Patients

We enrolled 57 consecutive MDS patients with IPSS or WPSS from intermediate to very high risk diagnosed between 2006 and 2019 and treated with azacitidine (AZA) 75 mg/m^2^/day or 100 mg/m^2^/day via subcutaneous injection or IV infusion. Of the whole cohort, 42 cases were diagnosed and studied at Policlinico *Maggiore* di Milano, while the remaining 15 were provided by IRCCS Policlinico *Sant’Orsola* of Bologna. For each patient, clinical data, treatment duration, and follow-up data were collected together with morphological and phenotypical features of BM biopsies performed within 8 weeks prior to treatment and any time after 3 to 12 months of treatment. For each patient, blast counts evaluated by flow-cytometry analysis on BM aspirate and on peripheral blood were also collected. Response to therapy was evaluated according to the 2006 IWG guidelines [14]. For the purpose of the study, we considered patients with partial and complete remission, hematological improvement, and stable disease as responders to azacitidine therapy. We also recorded the best overall response, defined as the best response obtained from the start of treatment until disease progression or recurrence. Progression to AML was considered when blasts count was ≥20% in bone marrow or peripheral blood. Six patients (10%) received hematopoietic stem cell transplantation; of these, 2 patients reached complete remission, two patients reached partial remission and 2 patients showed stable disease.

### 2.2. Bone Marrow Morphologic Evaluation

Each BM biopsy was stained with hematoxylin-eosin, Giemsa, and Gomori’s silver impregnation. The following morphological variables concerning diserytropoiesis were evaluated: megaloblastoid changes (elements characterized by at least 1.5 times the size of a normal proerytroblast with finely dotted chromatin and increase of the nucleus-cytoplasmic ratio), left-shifting, cytoplasmic vacuolization, nuclear alteration (including budding and multinuclearity) and topographic abnormalities. To define dysgranulopoiesis, nuclear hypo- or hyper-segmentation and left-shifting together with the presence of abnormal localization of immature precursors (ALIP) were considered, while the presence of micromegakaryocytes (mononuclear elements with a nuclear diameter of 7–10 μm), megakaryocytes with hypolobated nuclei and/or multinucleated ones and topographic abnormalities were quantified to assess the megakaryocyte dysplasia. Each specific alteration was carefully investigated by reviewers and considered as present if clearly identifiable along the entire specimen. Other parameters collected were the overall BM cellularity in relation to patient’s age, the myeloid/erythroid (M/E) ratio, blast count, and entity of marrow fibrosis (determined according to the EUMNET consensus [15]). Percentage assessment of immunohistochemical p53 positive (Dako Omnis DO-7) cells, CD34 positive (Ventana 790-2927) blasts, CD20 (Dako OMNIS L26), and CD3 positive (Dako OMNIS Polyclonal antibody) lymphocytes were performed using the automatic system BenchMark XT (Ventana Medical Systems). Reactions were revealed using the UltraViewTM Universal DAB, a biotin-free, multimer-based detection system, according to the manufacturer’s instruction. Immunohistochemical quantitative p53+ expression evaluation was performed among the entire specimen, considering positive only cells with intense nuclear expression of p53 (3+), as previously published [16,17,18,19]. The lymphocytic infiltrate was described as composed mainly of T lymphocytes or by a mixed T and B-cell population, quantified and defined if present in nodules, micro-aggregates, or with an interstitial pattern. MDS subtypes were defined according to the World Health Organization (WHO) 2017 criteria. We did not perform NGS or FISH studies on our cohort.

### 2.3. Statistical Analysis

Statistical analysis was performed with IBM SPSS Statistics, version 24.0 (IBM Corp., Armonk, NY, USA). Statistical significance was defined as *p* < 0.05. Distribution normality was assessed with the Shapiro–Wilk test. Overall survival was calculated from the start of AZA treatment until the time of death or the last clinical follow-up. Other outcomes considered were the progression to LMA and response to treatment according to the IWG guidelines. Survival curves were generated using the Kaplan–Meier method, and differences were evaluated using the log-rank test. Multivariate binary logistic regression analysis was constructed considering the endpoint and the outcome as a dependent variable. Independent sample *t*-tests were also conducted in order to compare the mean value of the continuous variables in the two subgroups of patients are stratified based on their outcomes. Finally, Pearson analysis was run in order to evaluate the potential correlation between different values.

## 3. Results

Clinical and prognostical features of the cohort evaluated are listed in Table 1. 

**Figure 1:**
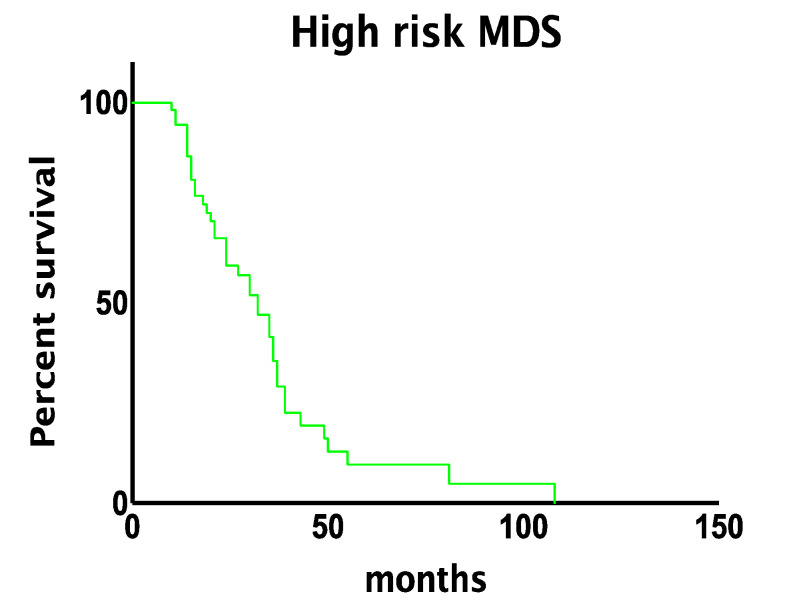
Median overall survival of high-risk myelodysplastic syndromes.

High-risk MDS showed a slight male predominance (M/F ratio = 1.59). At the time of diagnosis, most cases belonged to the WHO category of myelodysplastic syndromes with excess blasts (53/57, 93% of cases, with 41/53, 77% represented by MDS-EB type 2), with a minority (4/57, 7% of cases) diagnosed as myelodysplastic syndrome with multilineage dysplasia. After a median follow-up of 23 months and a median number of 13 cycles of azacitidine, 70% (40/57) of patients had died of disease, mostly due to AML evolution (28/57, 49% of cases), with a median time to leukemic progression of 17 months. The median overall survival was of 32 months (with a mean follow-up of 28 months, ranging from a minimum of five months to a maximum of 108 months, Figure 1).

According to IPSS, all MDS in our cohort belonged to the categories of intermediate and high risk, with the majority of cases (43/57, 75%) included in the intermediate risk, type 2 group. According to WPSS as well, all cases fit in the intermediate and high-risk categories, with the majority of cases (43/57, 75%) belonging to the high-risk group. When we applied the IPSS-R, we observed a prevalence of high-risk cases (28/57, 49%), followed by very high-risk cases (15/57, 27%), intermediate-risk cases (11/57, 19%), and a minority (3/57, 5%) of low-risk patients. We included these low-risk MDS in our series since their diagnosis was made prior to the introduction of IPSS-R and they were categorized as intermediate or high risk according to IPSS. IPSS-R cytogenetic scores were recorded before treatment for the whole series, while post-treatment best response scores were available only for 47 patients. In both cases, most MDS had a “good” cytogenetic score (47% pre-treatment and 60% post-treatment), followed by cases with “very poor” cytogenetic score (25% pre-treatment and 19% post-treatment). Lastly, according to IWG response criteria, most MDS showed complete remission (23/57, 41% of cases), while 20% of cases maintained stable disease (11/57, 20%) and 12% of patients had a hematological improvement; disease progression was observed in 14% of cases and 4% of patients failed to respond to chemotherapy. In our cohort, the best overall response was observed after a median number of 6 cycles of azacitidine.

### 3.1. Comparison between Pre-Treatment and Post-Treatment Morphological and Immunophenotypical Feature of BM Biopsies

Morphological and phenotypical features of pre- and post-treatment bone marrow biopsies are reported in Table 2. 

From a strictly morphological point of view, azacitidine treatment had no significant impact on bone marrow cellularity or dysplastic features of the three hemopoietic lineages. On the other hand, we observed a slight post-treatment increase in bone marrow lymphoid infiltrate, ranging from a pre-treatment median value of 7.63% to a post-treatment median value of 8.29%, similarly to what was previously reported by cytofluorimetry studies [20,21,22]. Inflammatory infiltrates showed an interstitial distribution with scattered microaggregates and they were mainly composed of T-cells, variably admixed with a minority of B-cells. We did not observe significant modifications in the number of mast cells or plasma cells. The number of CD34+ and CD117+ precursor elements evaluated on bone marrow biopsy showed a slight decrease after azacitidine treatment, varying from a median value of 12.63% to a median value of 11.11%. Similar observations were made regarding bone marrow aspirate smear, with a median decrease of 3.87% in blast count, and flow cytometry of bone marrow aspirate, with a median decrease of 1.27% in blast count. Surprisingly, peripheral blood smear showed a mean increase of 0.44% in blast count after azacitidine treatment in MDS-EB cases, especially in MDS-EB1 patients (recording a mean 1% increase in blast count). Obviously, however, single cases with partial and complete remission showed a blast decrease coherent with IWG response defining criteria. The number of p53+ elements was slightly decreased by azacitidine treatment, going from a pretreatment median value of 3.71% to a post-treatment median value of 3.10%, with a relevant reduction (from 2% to 0.38%) in MDS-MLD cases. Lastly, regarding bone marrow fibrosis, we observed a post-treatment reduction of MF-0 scores (from 51% to 48%) and MF-2 scores (from 17% to 14%) with parallel increase in MF-1 (from 32% to 33%) and MF-3 (from 0% to 5%). Moreover, higher degrees of bone marrow fibrosis (MF-2 and MF-3) evaluated before treatment were associated with lower OS at the multivariate model (*p* = 0.05) with an Odds Ratio of 2.491 of death from disease at any increasing point of fibrosis. Of note, none of the above-mentioned features showed statistically significant variations after azacitidine treatment.

### 3.2. Pre-Treatment Prognostic Factors

Pre-treatment IPSS-R cytogenetic score, lymphocytic infiltrate, and number of p53+ elements showed significant correlation either with response to therapy, AML progression, or both. Results are shown in Table 3.

### 3.3. Cytogenetic IPSS-R Score

As expected, cases with poor and very poor IPSS-R cytogenetic scores evaluated at diagnosis and before treatment demonstrated a higher probability of leukemic progression (*p* = 0.004). Interestingly, higher post-treatment cytogenetic scores correlated proportionally with myelofibrosis degree encountered in BM biopsy, with a proportionally higher number of MF-2 (12% in high-risk cases vs. 10% in low-risk cases) and MF-3 cases (6% in high-risk cases vs. 3% in low-risk cases). Poor and very poor pre- and post-treatment IPSS-R scores were also related to a higher number of pre- and post-treatment p53-positive elements. Among pre-treatment IPSS-R low-risk MDS, 9/37 cases (24%) had >1% of p53+ elements (ranging from 1% to 5%) while in the high-risk group, the number of cases was higher (11/20, 55%), with p53+ elements ranging from 1% up to 40%. Similar results were obtained for post-treatment bone marrow biopsies, with 17% (9/35) of cases showing >1% p53+ elements (ranging from 1% to 15%) among low-risk cases versus 50% (6/12) of cases with up to 40% p53+ elements in high-risk cases.

### 3.4. Lymphocytic Infiltrate

Response to treatment was reached more frequently in patients with higher lymphocytic infiltrate in BM biopsy performed before treatment (*p* = 0.004), with a mean percentage of lymphoid cells among responders of 8.21% and of 4.9% among non-responders. Similarly, patients with a higher percentage of lymphocytes before treatment showed a lower probability of leukemic progression (*p* = 0.017) with a mean lymphocyte infiltrate of 6.64% among patients with subsequent progression and of 8.59% among non-progressed ones. This result was also confirmed by multivariate analysis (*p* = 0.05), with an 0.87 odds ratio of leukemic progression for each decreasing percentage point in lymphocytic infiltrate evaluated on pre-treatment bone marrow biopsy. A negative correlation according to the Pearson analysis was finally described between the presence of lymphocytic infiltrate, both in pre- and after-treatment biopsies, and after treatment blastic count performed on peripheral blood, bone marrow aspirate, and cytofluorimetry; an increase in the lymphocytic infiltrate corresponded to a parallel reduction in post-treatment blastic count.

### 3.5. p53-Positive Precursors

Quantification of pre-treatment positive elements in BM biopsies correlated with risk of progression to AML, with higher percentages of p53-positive elements before treatment (mean 4.7%) among progressed patients (*p* = 0.001) compared to the ones without progression (mean 0.8%). We also observed a positive linear correlation, according to Pearson analysis, between p53-positive count in pre-treatment biopsy and blastic amount subsequently found in post-treatment biopsy; an increase of p53+ elements corresponded to higher post-treatment blastic count. Pre-treatment cases with ≥1% p53+ elements are described in Table 4.

### 3.6. Post-Treatment Prognostic Factors

Post-treatment blast count, lymphocytic infiltrate, and number of p53+ elements showed significant correlation either with overall survival, response to therapy, and/or AML progression. Results are shown in Table 5.

### 3.7. Blasts

**Figure 2:**
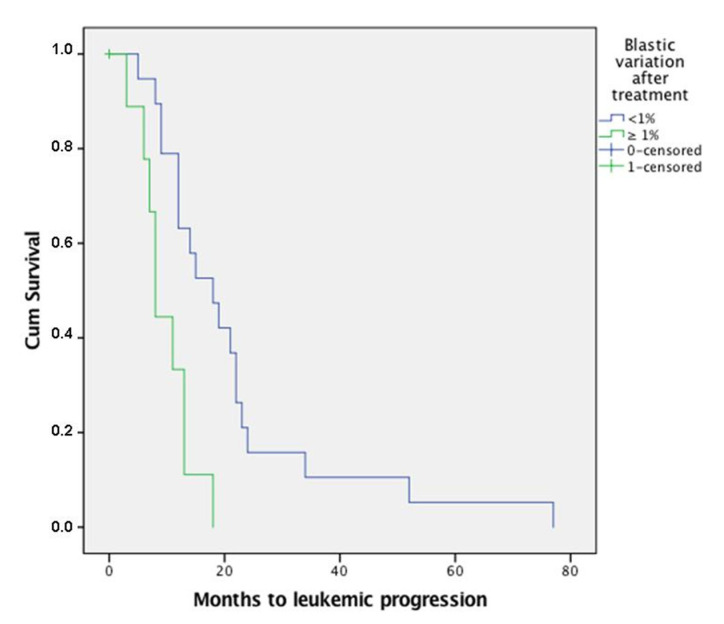
Progression to leukemic disease among patients with ≥1% increase in blast count after treatment occurred earlier than patients with no blast increase.

A higher post-treatment blast count portended inferior OS (*p* = 0.035), with a mean 11.7% blast count among patients who died of disease and of 8.29% among patients still alive at follow-up (mean follow-up of 28 months, ranging from 5 to 108 months). Higher blast count correlated also with progression to AML (*p* = 0.04), with a mean 12.4% blast count among patients with subsequent leukemic progression versus a mean value of 9.6% in non-progressed cases. Moreover, a post-treatment increase of at least 1% in bone marrow blast count was associated with a shorter progression to leukemic evolution (*p* = 0.002) (Figure 2).

### 3.8. Lymphocytic Infiltrate

Similar to pre-treatment bone marrow biopsies, higher lymphocytic infiltrates correlated with better response to treatment (*p* = 0.004), with mean values of lymphocytic infiltrate of 8.96% among responders and of 6.10% among non-responders.

### 3.9. p53-Positive Precursors

Likewise, also in post-treatment bone marrow biopsy the number of p53+ cells correlated with leukemic evolution, with a mean number of 3.2% p53+ elements among progressed patients compared to a mean number of 1.06% p53+ elements in non-progressed ones (*p* = 0.013). Finally, a lower number of post-treatment p53+ elements in BM biopsy was linked to better response to therapy (*p* = 0.003), with a mean value of 1.5% p53+ elements among responders versus 4.8% among non-responders.

## 4. Discussion

High-risk myelodysplastic syndromes represent a therapeutic challenge for clinicians, mainly due to patients’ age, performance status, and comorbidities at the time of diagnosis and during disease course. Hypomethylating agents, mainly represented by azacitidine, remain a milestone in treatment of high-risk MDS cases. However, to the best of our knowledge, no previous study has focused on modifications induced by azacitidine on bone marrow biopsy morphological and immunophenotypical characteristics. Our main purpose, as a consequence, was to evaluate whether azacitidine therapy significantly impacted any of bone marrow biopsy features. Our cohort showed a 62% rate of cumulative complete and partial response and hematological improvement to azacitidine, defined according to IWG criteria, reaching higher percentages than what was reported in AZA-001 study [7]. Response to therapy seemed to be associated with better overall survival, with a 64% death rate among responders and of 100% among non-responders, and 44.68% of progressed cases observed among responders compared to 70% in non-responders. The best overall response was obtained after a median number of 6 cycles of azacitidine. These results confirm that treatment duration should last at least six months, even in the absence of an early response to therapy, as suggested by other authors [23,24]. From the analyses conducted on bone marrow biopsies, we concluded that azacitidine does not induce significant modifications on bone marrow morphology. In fact, both responders and non-responders showed superimposable morphological features between pre- and post-treatment biopsies, with no regression of dysplastic features in any of the lineages affected. In this regard, the revised IWG criteria do admit the persistence of morphological dysplasia in the definition of complete response, with, however, the suggestion of noting its presence [14]. A recent study [25] has proposed some variations to IWG response criteria regarding erythroid response and hematological improvement, but it did not investigate bone marrow morphological features, which might be in need of revision in the next future in light of our results. Moreover, during the last years, bone marrow fibrosis has emerged as a significant prognostic and predictive factor in MDS, with bone marrow fibrosis grade 2 or higher being independently associated with poorer overall survival and, in some studies, with less consistent response to therapy [10,26,27]. Likewise, in our cohort, bone marrow fibrosis grade 2 or higher correlated with poorer outcome, confirming its prognostic relevance (*p* = 0.05). However, bone marrow fibrosis did not influence response to therapy. Besides, post-treatment modifications in bone marrow fibrosis were variable and not statistically significant. In addition, our study confirms the importance of the microenvironment in MDS, stating that pre- and post-treatment higher lymphocytic infiltrates correlate with better response to azacitidine, and that higher pretreatment lymphocytic infiltrates are linked to lower risk of AML progression. Of importance, only a slight, non-significant increase in lymphocytic infiltrate was observed after azacitidine treatment. The prognostic role of lymphocytic infiltrate is of particular interest and it has never been previously reported probably due to the fact that post-treatment evaluation mainly depends, in real-life settings, on peripheral blood and bone marrow aspirate evaluation and less frequently on bone marrow histology. Different studies have highlighted the relevance of bone marrow immune microenvironment in myelodysplastic syndromes, suggesting a possible mediated “autoimmune” etiopathogenetic role in some cases or a correlation between certain T-cell subtypes and disease aggressiveness, such as CD4+ and T-reg cells which may lead disease progression through a reduction of anti-neoplastic response [18]. Still, there is conflicting evidence circa the actual role of lymphoid infiltrates in MDS, since not all studies have confirmed their prognostic impact in MDS [11,21,28,29,30]. In our cohort, for instance, higher lymphocytic infiltrates (composed of a prevalence of interstitial and/or nodular CD3+ T-cells, variably admixed with a minority of CD20+ B-cells) were linked to better outcome and less frequent AML progression, suggesting a possible protective role of the reactive infiltrate from leukemic evolution. Unfortunately, since we did not perform an evaluation of T-cell subpopulations in bone marrow biopsies, a more complete picture of such correlation will need future phenotypical characterization. However, our findings highlight the promising role of post-treatment bone marrow histology as an integrative tool to peripheral blood and bone marrow aspirate evaluations, in order to reach a deeper insight into the MDS microenvironment and, thus, outcome. Azacitidine treatment did not have a statistically significant impact either on blast count and p53+ elements or on IPSS-R cytogenetic score. However, all the above-mentioned factors correlated either with disease progression, overall survival and/or response to therapy. Higher pre-treatment IPSS-R cytogenetic scores, in particular, showed a predictable correlation with AML progression (*p* = 0.004) but they also showed an unexpected, although not significant, correlation with pre-treatment bone marrow fibrosis and pre- and post-treatment number of p53+ elements, giving us another glimpse into the complexity of MDS pathogenesis. Higher bone marrow biopsy blast counts correlated with inferior OS and quicker disease progression; of interest, even the slightest increase of at least 1% in blast count after azacitidine was significantly (*p* = 0.002) associated with shorter progression to leukemic phase, suggesting the importance of a careful evaluation of blasts on bone marrow biopsies in order to properly identify cases with poorer prognosis. In conclusion, our study confirms the efficacy of azacitidine treatment, highlighting on the other hand, its lack of effects (at least in terms of statistically significant results) on bone marrow dysplasia or other features, such as lymphocytic infiltrate, number of p53+ elements, and blast count. Nevertheless, our results, although in need of future confirmation, might suggest reconsidering the role of bone marrow histology also in post-treatment evaluation, along with peripheral blood and bone marrow aspirate evaluation, and unveil the promising role of other tools as potential predictive markers of response and outcome, such as p53 expression and lymphocytic infiltrate.

## Figures and Tables

**Table 1:** Clinical and prognostic features of the cohort.

Sex (%)	
Female	22 (39%)
Male	35 (61%)
Follow up (mean value in months) (range)	28 (5–108)
Outcome (%)	
Dead of disease	40 (70%)
Alive with disease	17 (30%)
Diagnosis (%)	
MDS-EB1	12 (21%)
MDS-EB2	41 (72%)
MDS-MLD	4 (7%)
Number of AZA cycles (mean value) (range)	13 (3–41)
IWG response criteria—Best Response (%)	
Complete remission	23 (41%)
Partial remission	5 (9%)
Stable disease	11 (20%)
Hematological improvement	7 (12%)
Progression disease	8 (14%)
Failure	2 (4%)
Progression to AML (%)	28 (49%)
Months to progression, mean value (range)	17 (0–77)
IPSS (%)	
Intermediate-1 risk	4 (7%)
Intermediate-2 risk	43 (75%)
High risk	10 (18%)
IPSS-R (%)	
Low risk	3 (5%)
Intermediate risk	11 (19%)
High risk	28 (49%)
Very high risk	15 (27%)
WPSS (%)	
Intermediate risk	6 (11%)
High risk	43 (75%)
Very high risk	8 (14%)
IPSS-R Cytogenetic score	
Before treatment (57 pts)	
Very good	2 (4%)
Good	28 (47%)
Intermediate	7 (12%)
Poor	4 (7%)
Very poor	16 (25%)
After treatment (47 pts)—Best response	
Very good	2 (4%)
Good	28 (60%)
Intermediate	5 (11%)
Poor	3 (6%)
Very poor	9 (19%)

Abbreviations. MDS-EB1: myelodysplastic syndromes with excess blasts, type 1. MDS-EB2: myelodysplastic syndromes with excess blasts, type 2. MDSL-MLD: myelodysplastic syndromes with multilineage dysplasia. AML: acute myeloid leukemia. AZA: azacitidine. IPSS: internation prognostic scoring system. IPSS-R: international prognostic scoring system–revised. WPSS: WHO Classification-based Prognostic Scoring System.

**Table 2:** Comparison between pre- and post-treatment evaluation: blasts on bone marrow and detected on peripheral blood, aspirate smear and flow-cytometric, and other variables detected on bone marrow biopsy.

Bone Marrow Biopsy	Pre-Treatment Biopsy			Post-Treatment Biopsy		
Blasts	12.62%			11.11%		
	MDS-EB1 6.42%	MDS-EB2 15.33%	MDS-MLD 3.5%	MDS-EB1 12.42%	MDS-EB2 10.37%	MDS-MLD 9.5%
p53	3.71%			3.1%		
	MDS-EB1 4.13%	MDS-EB2 2.71%	MDS-MLD 0.5%	MDS-EB1 3.11%	MDS-EB2 2.08%	MDS-MLD 0.5%
Lymphocytic infiltrate	7.63%			8.29%		
	MDS-EB1 7%	MDS-EB2 8%	MDS-MLD 4%	MDS-EB1 6%	MDS-EB2 9%	MDS-MLD 6%
Fibrosis						
MF-0	29 (51%)			27 (48%)		
	MDS-EB1 58.33%	MDS-EB2 46.4%	MDS-MLD 75%	MDS-EB1 41.66%	MDS-EB2 51.21%	MDS-MLD 25%
MF-1	18 (32%)			19 (33%)		
	MDS-EB1 8.33%	MDS-EB2 39%	MDS-MLD 25%	MDS-EB1 16.66%	MDS-EB2 34.14%	MDS-MLD 75%
MF-2	10 (17%)			8 (14%)		
	MDS-EB1 33.33%	MDS-EB2 14.6%	MDS-MLD 0%	MDS-EB1 25%	MDS-EB2 12.19%	MDS-MLD 0%
MF-3	0 (0%)			3 (5%)		
	MDS-EB1 0%	MDS-EB2 0%	MDS-MLD 0%	MDS-EB1 16.66%	MDS-EB2 2.43%	MDS-MLD 0%
Blasts on peripheral blood	2.19%			2.63%		
	MDS-EB1 2%	MDS-EB2 2%	MDS-MLD 0%	MDS-EB1 4%	MDS-EB2 3%	MDS-MLD 0%
Blasts on aspirate smear	11%			6.71%		
	MDS-EB1 7%	MDS-EB2 12%	MDS-MLD 3%	MDS-EB1 5%	MDS-EB2 8%	MDS-MLD 4%
Blasts on flow-cytometry	5.62%			4.35%		
	MDS-EB1 8%	MDS-EB2 8%	MDS-MLD 0%	MDS-EB1 6.7%	MDS-EB2 5.3%	MDS-MLD 0%

Abbreviations. MDS-EB1: myelodysplastic syndromes with excess blasts, type 1. MDS-EB2: myelodysplastic syndromes with excess blasts, type 2. MDSL-MLD: myelodysplastic syndromes with multilineage dysplasia. MF: myelofibrosis, graded according to to the EUMNET consensus [15].

**Table 3:** Pre-treatment biopsy prognostic factors.

	Overall Survival	Progression to AML	Response to Treatment
Higher R-IPSS cytogenetic risk	-	Positive correlation; *p* = 0.004 (poor/very poor risk in AML: 46%; poor/very poor risk in non-AML: 24%)	-
Higher lymphocytic infiltrate	-	Negative correlation; *p* = 0.017 (mean percentage in AML: 6.64; mean percentage in non-AML: 8.59)	Positive correlation; *p* = 0.004 (mean percentage in responders: 8.21; mean percentage in non-responders: 4.9)
Higher p53 expression	-	Positive correlation; *p* = 0.001 (mean percentage in AML: 4.7; mean percentage in non-AML: 0.8)	-

Abbreviations. AML: acute myeloid leukemia. IPSS-R: international prognostic scoring system—revised.

**Table 4:** Cases with ≥1% p53+ elements. Only pre-treatment bone marrow biopsies were considered.

Number of Cases	20/57 (35%)
Mean percentage of p53+ elements (range)	6% (1–40%)
IPSS	
Intermediate-1 risk	0
Intermediate-2 risk	16/20 (80%)
High risk	4/20 (20%)
IPSS-R	
Low risk	2/20 (10%)
Intermediate risk	3/20 (15%)
High risk	7/20 (35%)
Very high risk	8/20 (40%)
WPSS	
Intermediate risk	0
High risk	15/20 (75%)
Very high risk	5/20 (25%)
Mean number of AZA cycles (range)	6 (3–9)
Cytogenetic Score IPSS-R	
Very good	0
Good	7/20 (35%)
Intermediate	2/20 (10%)
Poor	1/20 (5%)
Very poor	10/20 (50%)

Abbreviations. AZA: azacitidine. IPSS: internation prognostic scoring system. IPSS-R: international prognostic scoring system–revised. WPSS: WHO Classification-based Prognostic Scoring System.

**Table 5:** Post-treatment biopsy.

	Overall Survival	Progression to AML	Response to Treatment
Higher blastic count	Negative correlation; *p* = 0.035 (mean percentage in dead of disease: 11.7; mean percentage in alive at follow up: 8.29)	Positive correlation; *p* = 0.04 (mean percentage in AML: 12.4; mean in non-AML: 9.6)	-
Higher lymphocytic infiltrate	-	-	Positive correlation; *p* = 0.004 (mean percentage in responders: 8.96; mean percentage in non-responder: 6.10)
Higher p53 expression	-	Positive correlation; *p* = 0.013 (mean percentage in AML: 3.2; mean percentage in non-AML: 1.06)	Negative correlation; *p* = 0.003 (mean percentage in responders: 1.5; mean percentage in non-responders: 4.8)

AML: acute myeloid leukemia.

## Data Availability

The data presented in this study are available on request from the corresponding author.
